# High-Efficient and Recyclable Magnetic Separable Catalyst for Catalytic Hydrogenolysis of β-O-4 Linkage in Lignin

**DOI:** 10.3390/polym10101077

**Published:** 2018-09-28

**Authors:** Jingtao Huang, Chengke Zhao, Fachuang Lu

**Affiliations:** 1 State Key Laboratory of Pulp and Paper Engineering, South China University of Technology, Guangzhou 510640, China; 201620121261@mail.scut.edu.cn (J.H.); mszck@foxmail.com (C.Z.); 2 Guangdong Engineering Research Center for Green Fine Chemicals, Guangzhou 510640, China

**Keywords:** lignin, magnetic catalyst, palladium, hydrogenolysis, β-O-4

## Abstract

Lignin is recognized as a good sustainable material because of its great abundance and potential applications. At present, lignin hydrogenolysis is considered as a potential but challenging way to produce low-molecular-mass aromatic chemicals. The most common linkage between the structural units of lignin polymer is the β-O-4 aryl ether, which are primary or even only target chemical bonds for many degradation processes. Herein, a Pd-Fe_3_O_4_ composite was synthesized for catalytic hydrogenolysis of β-O-4 bond in lignin. The synthesized catalyst was characterized by XRD, XPS, and SEM and the lignin depolymerization products were analyzed by GC-MS. The catalyst showed good catalytic performance during the hydrogenolysis process, lignin dimer was degraded into monomers completely and a high yield of monomers was obtained by the hydrogenolysis of bagasse lignin. More importantly, the magnetic catalyst was separated conveniently by magnet after reaction and remained highly catalytically efficient after being reused for five times. This work has demonstrated an efficient & recyclable catalyst for the cleavage of the β-O-4 bond in lignin providing an alternative way to make better use of lignins.

## 1. Introduction

As the second largest component of Lignocellulose materials, lignin is a renewable and sustainable natural polymer with great potential applications [1,2,3]. Ten to thirty percent of the mass and 40% of the energy in lignocellulosic biomass are made up of lignin [4]. However, lignin is usually used as combustion material to generate heat and electricity and less than 5% lignin has been used for other purpose nowadays, which obviously is not a desirable way for lignin to be utilized [5,6]. From the perspective of the chemical structure of lignin and its potential applications, it was suggested that lignin can be a great source of valuable aromatic chemicals if the natural lignin could be broken into small molecular units [7]. However, the most difficult aspect of using this technique was that the depolymerization process for such conversions of lignocellulose materials has been very elusive [8].

Lignins are amorphous polymers constituted of several basic units, it can be regarded as a cross-linked macromolecule derived from the phenylpropanoid compound, coniferyl alcohol, and related alcohols, with the proportions depending on the plant species [9,10]. The interconnections between phenylpropane units are various C–O linkages (C–O–C=β–O–4, α-O-4, 4-O-5 etc.) and C–C interunit linkages [11,12]. Amongst linkages between structural units, the β-O-4 ether bond occupies the majority of the linkages, which makes up a 50% proportion in the softwood lignin and up to 60% proportion in hardwood [13]. Therefore, how to cleave the β–O–4 bond completely during the depolymerization of lignin is the critical point for lignin degradation [14].

Many methods for lignin depolymerization have been reported including hydrolysis, oxidation methods, and reduction methods. Hydrolysis of C–O–C linkages in lignin leads to phenol derivatives’ products but yield is quite low, the oxidation method involves oxidative cleavage of C–H bonds and/or C–C bonds adjacent to C–O–C linkages, and these reactions usually increase the already high oxygen content of the material and is therefore less attractive [15]. The reductive method is thought to be a promising method of lignin depolymerization to phenolic monomers. One of the popular reductive methods for lignin depolymerization is hydrogenolysis [16,17]. Hydrogenolysis is a type of degradation reaction catalyzed by a hydrogenation catalyst under hydrogen conditions [18]. The Hydrogenolysis of lignin (reductive method) is one of the most prevalent and efficient strategies to produce aromatic chemicals from lignin [19]. During the hydrogenolysis process, a metallic catalyst is usually used to increase the selectivity of hydrogenolysis and lower the reaction activation energy [20,21]. Among the other catalysts, heterogeneous Pd-based catalyst system has been deemed to be an efficient catalyst for hydrogenolysis of β-O-4 linkages of lignin model compounds and lignins [22,23,24]. However, the disadvantage of this catalyst is that the palladium is rare and expensive and the palladium catalyst is hard to be separated and recycled after the reaction [25]. Therefore, from the perspective of cost and efficiency, it is very essential to avoid Pd leaching during reactions and increase the recycle times of using the catalyst. A heterogeneous Pd-based catalyst could meet the requirement of high reaction efficiency without Pd’s leaching and reclamation difficulty. 

When the noble metal atom is deposited onto the surface of magnetic materials, a unique catalyst can be made as it can be separated easily after reaction by applying a magnetic field [26,27,28]. Hence, the magnetic noble metal catalyst can be recycled many times without the leaching of noble metals improving the catalytic efficiency and life spam of the catalyst. A wide range of magnetic materials, including metallic materials and metalloid materials, have been found. Ferric oxide and nickel oxides are the most common magnetic materials [29,30]. Being an important spinellide ferric oxide, Fe_3_O_4_ is one of the most widely used magnetic materials used as recording materials, pigment, catalyst, electrical materials, etc. [31,32,33]. Nowadays, Fe_3_O_4_ has found applications in chemical reaction and biomedicine research as an excellent magnetic material [34,35,36]. Wuang et al. synthesized polypyrrole-Fe_3_O_4_ nanoparticles for application in biomedicine, and alcohol oxidation was performed under ferrite magnetic nanocatalyst by Bhat et al. [36,37], whereas the surface modified Fe_3_O_4_ may be indispensable for further application. Owing to the magnetism of the magnetic materials, the magnetic material offers a new application in the catalytic yield. Functionalized magnetic particles are heterogeneous catalyst-supports, which have emerged as better alternatives compared to conventional materials because they are robust, inert, inexpensive, reusable, and recyclable by using a simple magnet [37,38]. A series of Pd-Fe catalysts and Pd-Fe_3_O_4_ catalyst had been reported for catalytic cleavage of C–O bond in lignin model compounds like α-O-4 model molecule, 4-O-5 model molecule, and β-O-4 model molecule. [39] The hydrogenolysis of lignin dimer by Pd-Fe or Pd-Fe_3_O_4_ was performed, under H_2_ as a reducing agent, and reaction temperature was above 200 °C. 

In current work, we have synthesized a Fe_3_O_4_ magnetic material functionalized with Pd^0^, which is used for the hydrogenolysis of β-O-4 lignin model compounds and bagasse lignin. The co-precipitation method was used to fabricate Fe_3_O_4_ magnetic particles and the Pd-Fe_3_O_4_ was then prepared by a simple wet impregnation method followed by a chemical reduction. Using the obtained Pd-Fe_3_O_4_ as a catalyst under a mild reaction condition (150 °C) with HCOONa as the reducing agent to provide the hydrogen and ethanol as a solvent, the lignin dimer and bagasse lignin were converted completely because the catalyst showed excellent catalytic performance. The results from this study indicated that the magnetic Pd-Fe_3_O_4_ catalyst would provide a viable option for the production of aromatic monomers from lignin via a catalytic hydrogenolysis.

## 2. Materials and Methods

### 2.1. Materials

FeCl_3_·6H_2_O (AR 99%), FeSO_4_·7H_2_O (AR) Urea (AR 99%), NaOH (GR 97%), PdCl_2_ (Pd 59–60%), NaBH_4_ (98%), Ethanol (AR), ethyl acetate (AR), monomers (monomer 2, H1, H2, H3, G1, G3, S1, S2, S3, S4) were purchased from Shanghai Macklin Biochemical Co., Ltd., (Shanghai, China). All chemicals were used as received without any further purification.

### 2.2. Synthesis of Fe_3_O_4_

5.4 g FeCl_3_·6H_2_O and 3.6 g Urea were dissolved in 200 mL of deionized water and stirred slowly to form a brown solution. Heat solution was set to 85 °C, stirring for 2 h. After reaction, the solution was cooled to room temperature. To the solution, 2.8 g FeSO_4_·7H_2_O was added, and then stirred to dissolve. 0.1 mol/L NaOH was drop-wise added to into the solution to pH = 10, and stirred for several minutes. After stirring, the suspension was transferred to an Ultrasonic bath, and sonicated for half an hour. After sonication, the solution was aged for 5 h. The obtained black precipitate was washed by water 3 times and was then washed by ethanol for 1 time. The Fe_3_O_4_ black precipitate was then dried in an oven at 55 °C for a night.

### 2.3. Synthesis of Pd-Fe_3_O_4_

Ferrite magnetic material Fe_3_O_4_ (2 g) and PdCl_2_ (0.34 g) were stirred at room temperature in water for (50 mL) for 1 h. After impregnation, the suspension was adjusted to PH 12 by adding NaOH (0.5 M) and stirred for 10 to 12 h. The solid was washed with distilled water (5 × 10 mL). The obtained metal precursors were reduced by adding 0.2 M aqueous NaBH_4_ until no bubbles were observed in the solution. The resulting Pd-Fe_3_O_4_ magnetic material was subjected to ultra-sonication for 10 min and then washed with distilled water and subsequently with ethanol and dried under oven at 60 °C for 24 h.

### 2.4. General Procedure for Lignin Model-Dimer Depolymerization Reaction

A mixture of ethanol, lignin model compound (4-benzyloxy-3-methoxyphenyl glycerol-β-aryl ether, 50 mg), HCOONa (200 mg), and magnetic catalyst (Pd-Fe_3_O_4_ 20 mg, the mol ratio of Pd atom and substrate is 14.1%) were sealed in an autoclave. The mixture was stirred in the autoclave at 150 °C for 6 h, with a stirring speed of 800 rpm. After reaction, the reaction solution was removed by simple decantation through using extra magnet to immobilize the catalyst on the bottom. Then the ethanol was evaporated and deionized water were added, the pH of reaction solution was adjusted to acidity by adding hydrochloric acid. At last, products were extracted by ethyl acetate.

### 2.5. General Procedure for Bagasse Lignin Extraction

50 g bagasse was put into a bottle with 400 mL NaOH solution (1.5 mol/L), and the mixture’s bottle was sealed and put into an oven with 90 °C for 1.5 h. Then, the liquid was filtered from mixture and the pH of filter liquid was adjusted to 6–7, triple volume of ethanol (95%) was added into the liquid with stirring, the obtained mixture was separated by centrifuge, and the liquid was collected. After using rotary evaporators to remove ethanol, the obtained concentrated solution was lignin’s solution. Adding HCL (1 mol/L) to adjust the pH of solution to 3, the suspension was centrifuged to separate the solid from mixture. At last, the lignin was obtained by freeze drying. The content of lignin is 90% analyzed by klason lignin. The extracted lignin was characterized by HSQC (for detail, see the supplementary information, Figures S1–S3).

### 2.6. General Procedure for Bagasse Lignin Depolymerization Reaction

Bagasse lignin (50 mg), HCOONa (500 mg), and magnetic catalyst (Pd-Fe_3_O_4_ 20 mg) and a mixture of ethanol (80%) and deionized water (20%) were sealed in an autoclave. The mixture was stirred in the autoclave at 150 °C for 6 h, with stirring speed of 800 rpm. After reaction, the reaction solution was removed by using extra magnet to immobilize the catalyst on the bottom. Then the ethanol and water were evaporated and deionized water was added. Then, the pH of reaction solution was adjusted to acidity by adding hydrochloric acid. The organic solvent phase was isolated and evaporated to obtain the products. At last, products were extracted by ethyl acetate.

### 2.7. Catalyst Characterization

These magnetic materials’ morphologies and structures of catalysts were characterized by scanning electron microscopy (SEM, LEO 1530 VP, Zeiss, Shanghai, China). X-ray photoelectron spectroscopy (XPS) was conducted on a Kratos Axis Ulra DLD (Kratos, Shanghai, China). X-ray powder diffraction (XRD) patterns of samples were performed on a Bruker D8 ADVANCE (Bruker, Beijing, China). Pd element’s weight percentage was estimated by AAS (Atomic Absorption Spectrometer, Z-2000, Hitach, Guangzhou, China).

### 2.8. Analytical Methods

The analysis of lignin model dimer and monomer products were carried out on GCMS-TQ8040 (Shimadzu, Shanghai, China) equipped with an SH-Rxi-5Sil column. The injection temperature was 250 °C, and the column temperature program was 50 °C (2 min) and 15 °C/min to 300 °C (15 min). The detection temperature was 250 °C. The products were well-separated by a capillary column. The contents of the monomer product in the samples were calculated by the internal standard method, the internal standard is dodecane.

(1) $$\mathrm{Yield}\;\mathrm{of}\;\mathrm{monomers}=\frac{\mathrm{weight}\;\mathrm{of}\;\mathrm{monomers}\;\mathrm{produced}}{\mathrm{weight}\;\mathrm{of}\;\mathrm{reactant}}\times 100\%$$

### 2.9. Recycling of the Catalysts

After all the reaction, the Pd-Fe_3_O_4_ catalyst was separated from the mixture by an external magnet, washed 3 times by ethanol and dried in a vacuum oven at 60 °C overnight. The collected catalyst was reused for the next cycle under the identical reaction conditions.

## 3. Results and Discussions

### 3.1. Preparation and Characterization of the Pd-Fe_3_O_4_ Catalyst

The Pd-Fe_3_O_4_ magnetic catalyst was prepared by two facile experimental steps, including co-precipitation and wet-impregnation [24]. Fe_3_O_4_ magnetic particles were first obtained from FeCl_3_·6H_2_O, FeSO_4_·7H_2_O in Urea solution through co-precipitation by adjusting the pH values of the solution with NaOH. Then, the Fe_3_O_4_ particles were impregnated in a PdCl_2_ solution with stirring. The resultant mixture was reduced by NaBH_4_ to immobilize Pd^0^ on the surface of Fe_3_O_4_ particles to produce the magnetic Pd-Fe_3_O_4_ catalyst.

**Figure 1:**
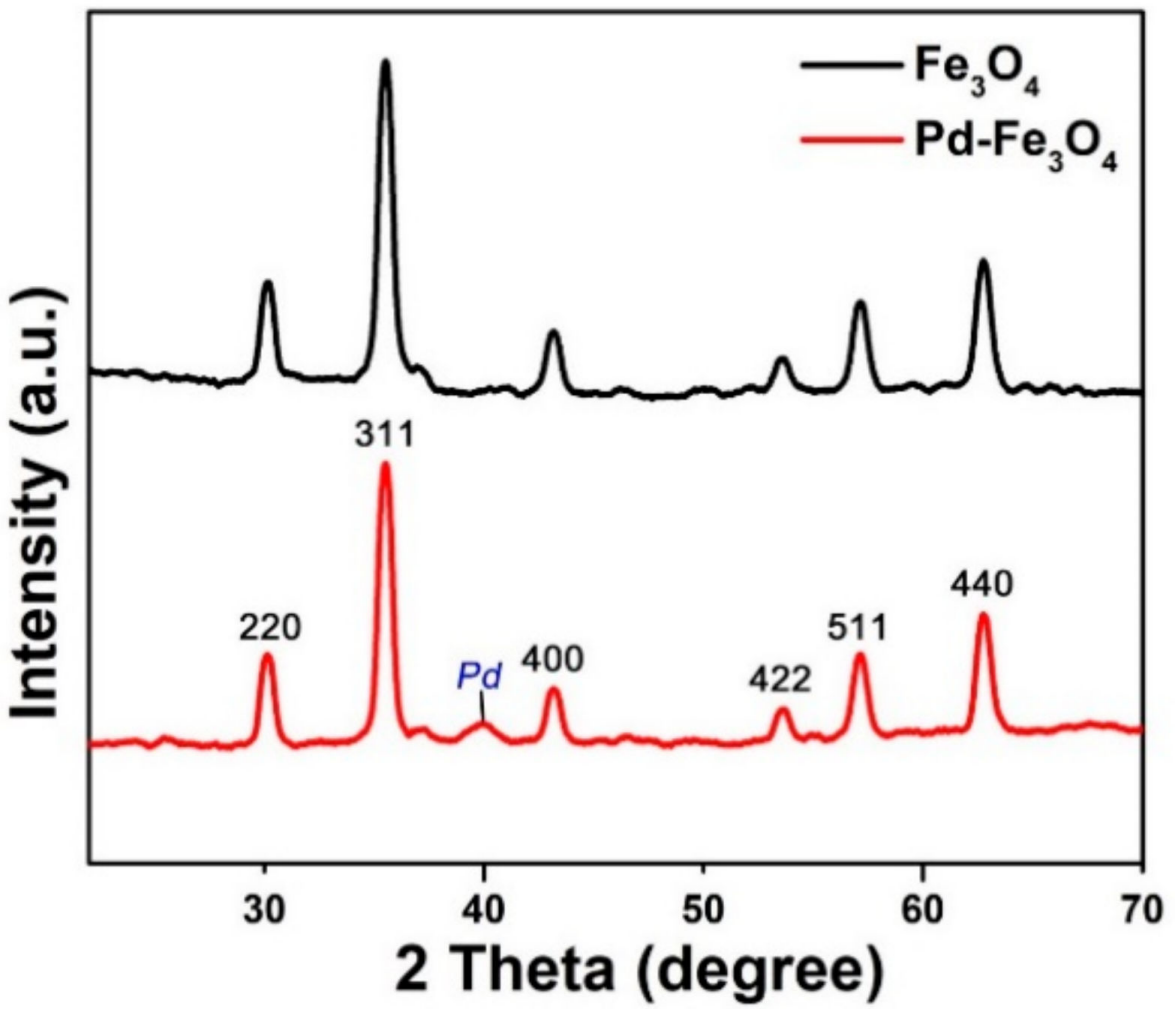
XRD patterns of Fe_3_O_4_ and Pd-Fe_3_O_4._

The crystalline structures of the resulting products (Fe_3_O_4_ and Pd-Fe_3_O_4_) were investigated by XRD. In Figure 1, several peaks of the Pd-Fe_3_O_4_ composites were found to be similar to the Fe_3_O_4_ sample. The characteristic diffraction peaks in the samples at 2θ at 30.1°, 35.5°, 43.3°, 53.8°, 57.1°, and 62.8°, correspond to the diffraction of (220), (311), (400), (422), (511), and (440) of Fe_3_O_4_ [25]. All the diffraction peaks matched with a magnetic cubic structure of Fe_3_O_4_ and the distinct and strong peaks confirmed that the products were well crystallized. More importantly, a new peak that appeared at 2θ = 39.8° was attributed to the Pd species, implying that the Pd atoms have been immobilized on the surface of Fe_3_O_4_.

**Figure 2:**
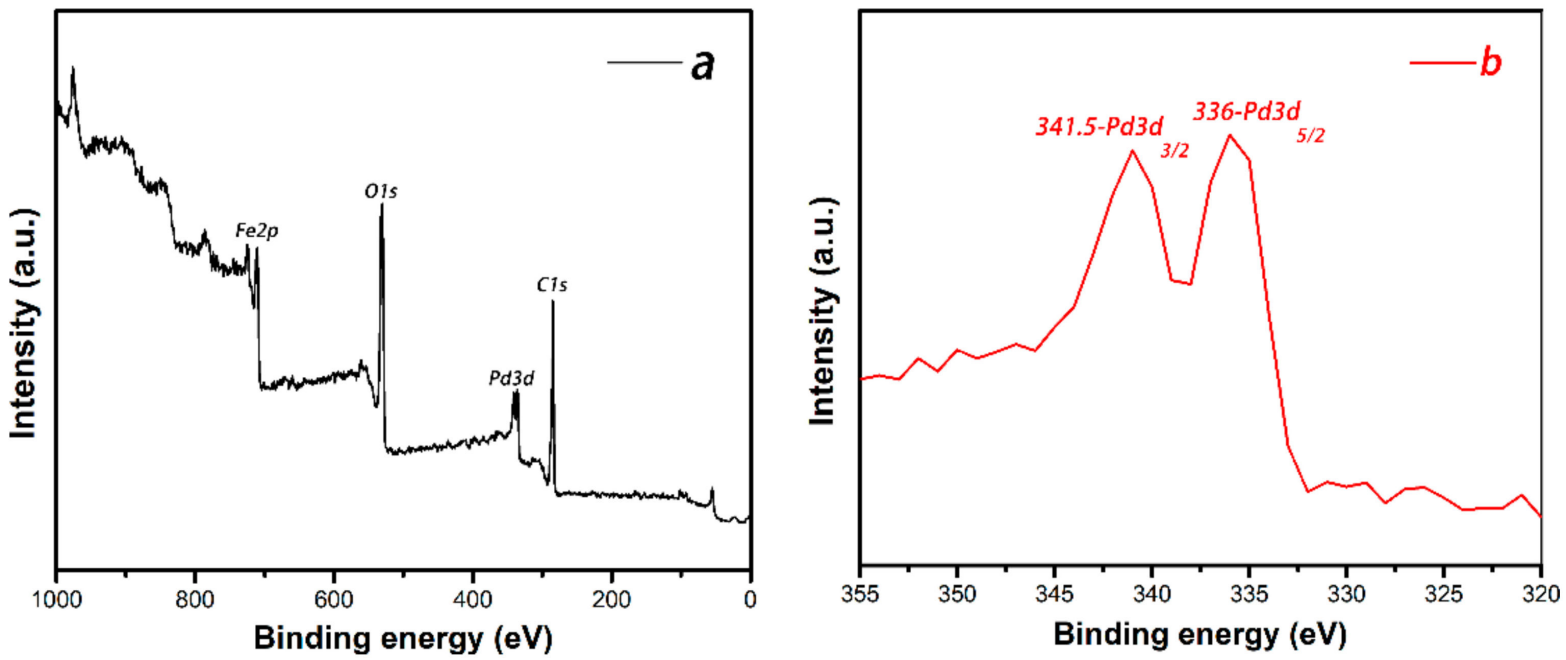
(**a**): XPS spectrum of Pd-Fe_3_O_4_. (**b**): XPS spectrum of Pd^0^ in Fe_3_O_4_.

XPS of the Pd-Fe_3_O_4_ confirmed the existence of Pd atom and its zero covalent state. Figure 2 presents an XPS elemental survey scan of the surface of Pd-Fe_3_O_4_ catalyst. In Figure 2a, the peaks corresponding to oxygen (530.2 eV), carbon (285.1 eV), palladium (335.5 eV), and iron (710.5 eV) were clearly observed. These values correspond to the binding energy of the four compositional elements for the catalyst, indicating the Pd atom’s existence in Pd-Fe_3_O_4_. The binding energy of Pd 3d5/2 and Pd3/2 for Pd-Fe_3_O_4_ was found to be 341.5 and 336 eV in Figure 2b, which were in agreement with the binding energy of Pd^0^ in the composite.

**Figure 3:**
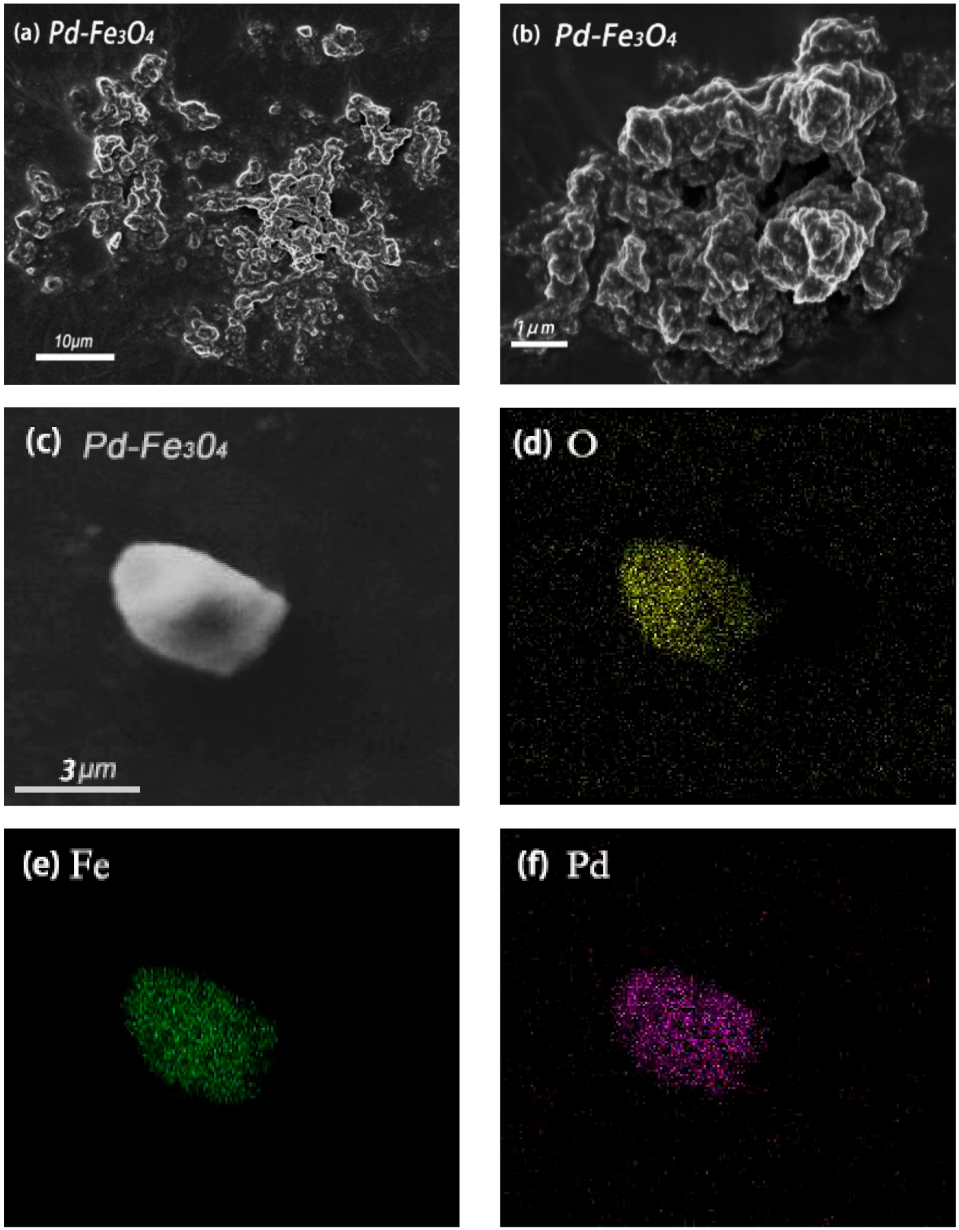
(**a**,**b**): SEM image of Pd-Fe_3_O_4_, (**c**–**f**): corresponding EDX mapping image of O, Fe and Pd.

Figure 3 shows the SEM pictures and EDX mapping images of the synthesized Pd-Fe_3_O_4_ particles, Figure 3a,b are the typical SEM images of Pd-Fe_3_O_4_, and EDX mapping images were showed in Figure 3c–f. As we can see from Figure 3a,b, the SEM pictures show that the morphology of magnetic particles were non-spherical & irregular particles with sizes at the micron level. The diameters of the magnetic particles were various with an average size of 3–8 microns while some particles were aggregated to bigger agglomerates. Figure 3b shows that the surfaces of Pd-Fe_3_O_4_ were rough, and the irregular rough surface may increase the particles’ surface area so as to improve palladium’s adhesive rate. Figure 3c–f shows the SEM pictures of a signal particle and the corresponding EDX elemental mapping of O, Fe, and Pd. The purple bright region in Figure 3f denotes the palladium’s existence on Pd-Fe_3_O_4_’s surface and the Pd^0^ was distributed on the particle’s surface homogeneously, like the purple dots. According to the AAS analysis, the Pd atom’s weight percentage in Pd-Fe_3_O_4_ is 8.54%.

### 3.2. Catalytic Hydrogenolysis of β-O-4 Model Compound and Bagasse Lignin by Pd-Fe_3_O_4_

**Scheme 1:**
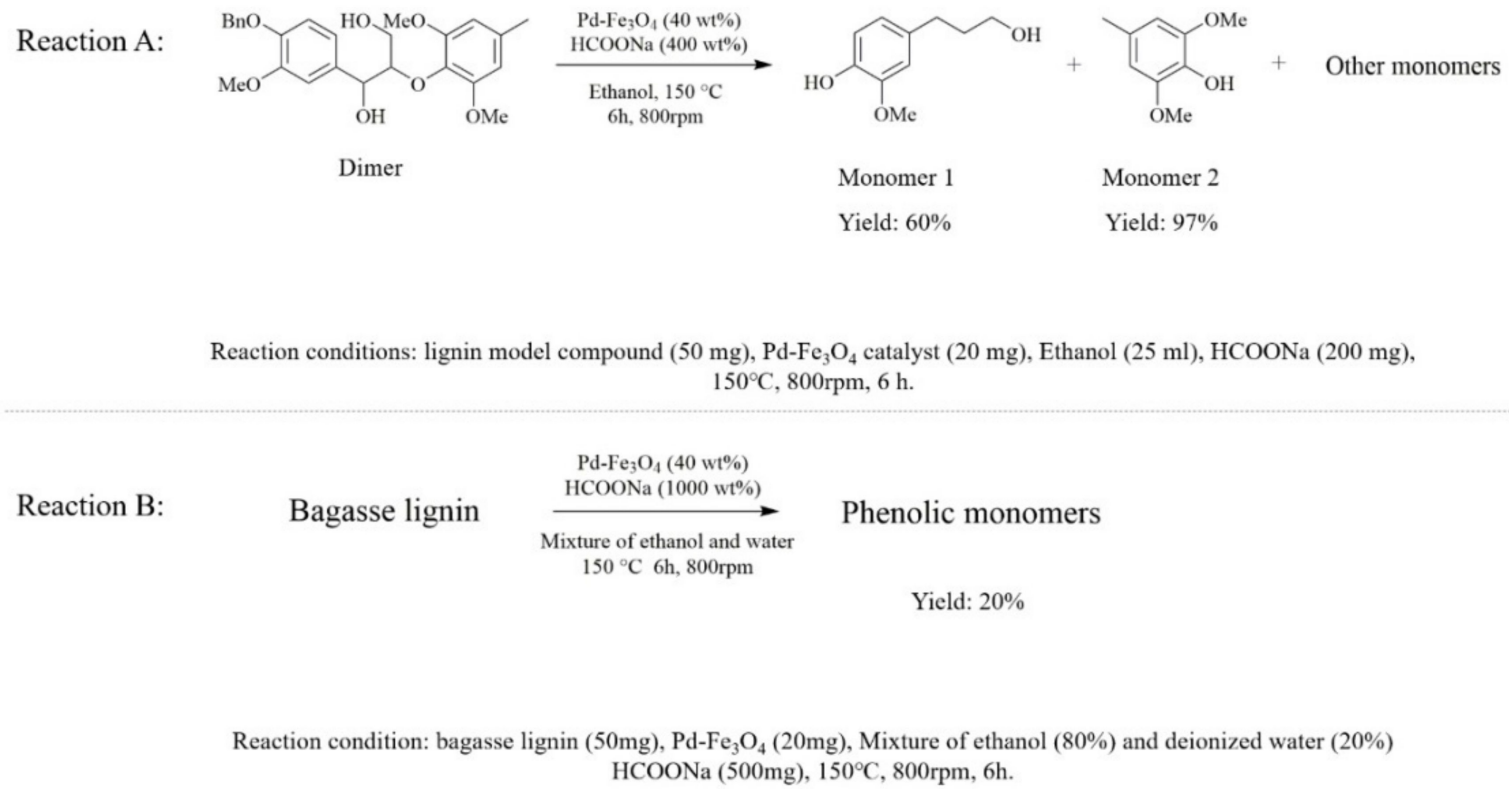
Catalytic hydrogenolysis of lignin model compound and bagasse lignin over Pd-Fe_3_O_4_ magnetic catalyst.

To explore the potential of Pd-Fe_3_O_4_ as a catalyst for the catalytic hydrogenolysis of β-O-4 linkages in lignin, a β-O-4 model compound and bagasse lignin, isolated from bagasse through the treatment of mild alkaline extraction, were used as substrates to perform the hydrogenolysis reaction (Scheme 1).

**Figure 4:**
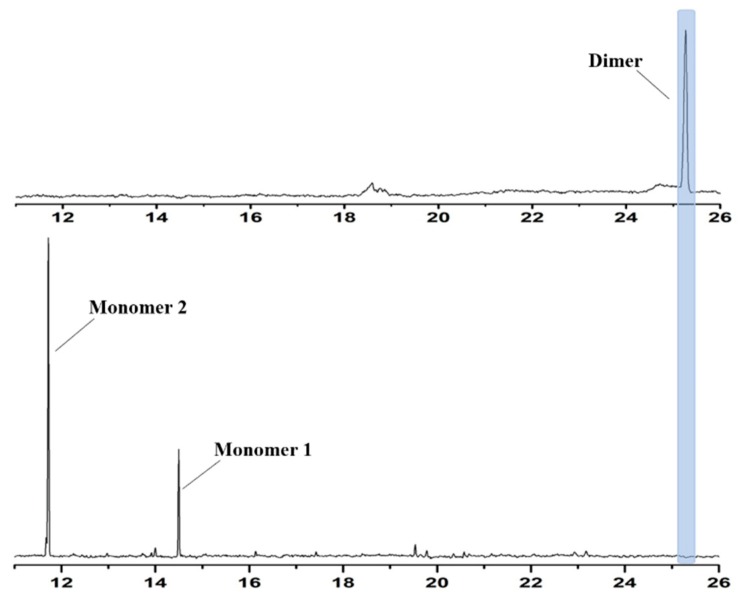
Gas chromatogram comparison of the lignin model compound and catalytic hydrogenolysis products over Pd-Fe_3_O_4_ magnetic catalyst.

On treatment of lignin model dimer with Pd-Fe_3_O_4_ and excess HCOONa in ethanol at 150 °C for 6 h in an autoclave, the catalyst was separated easily by a magnet and the reaction’s products in brown solution is recovered by evaporation under reduced pressure. The monomer products were extracted by ethyl acetate and analyzed by GCMS analysis (Figure 4). The hydrogenolysis products were identified by comparing their GC retention times and mass spectra with those of the authentic reference compounds acquired from commercial purchase or independent synthesis (for detail, see the supplementary information). In Figure 4, no starting dimer was detected in the hydrogenolysis products of dimer catalyzed by Pd-Fe_3_O_4_, indicating the lignin model dimer was converted completely. We also used Pd/C for lignin model dimer’s hydrogenolysis in the same reaction condition, the dimer depolymerized completely, too. The Pd-Fe_3_O_4_ showed equal catalytic performance compared with Pd/C in the same reaction condition for catalytic hydrogenolysis of β-O-4 linkage.

Two main products were found at the retention time of 12 and 14 min, referring to monomer 1 and 2, respectively. These results indicated the complete cleavage of β-O-4 bond in dimer after the catalytic hydrogenolysis of β-O-4 lignin model dimer by magnetic Pd-Fe_3_O_4_ catalyst.

**Figure 5:**
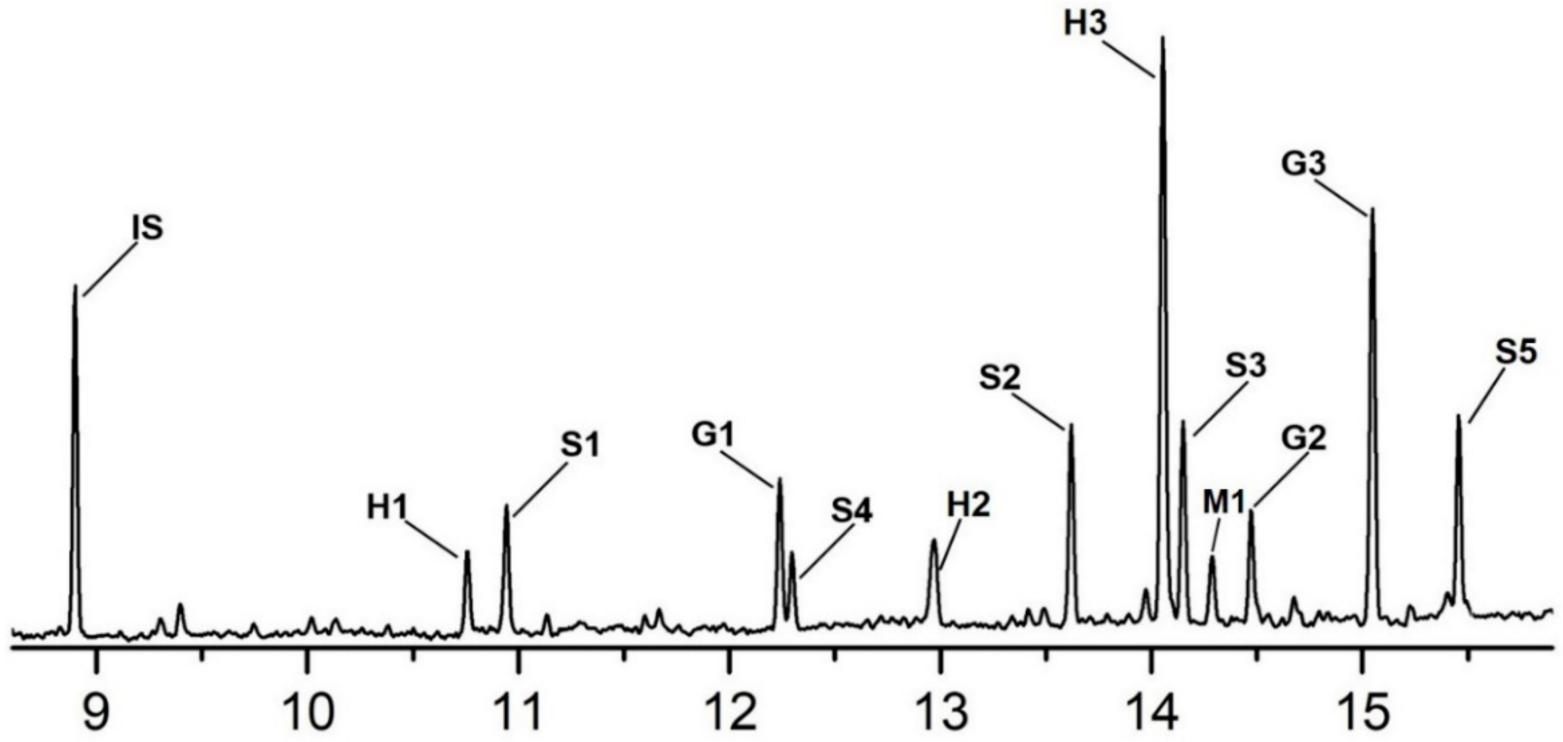
Total ion chromatogram and peak identification of the lignin monomers released from catalytic hydrogenolysis of bagasse lignin over Pd-Fe_3_O_4_ (reaction condition from Scheme 1, M1 = monomer 1).

**Figure 6:**
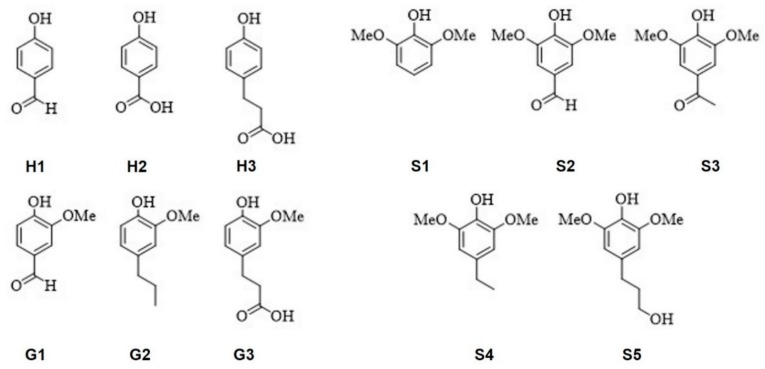
Summary of monomer products obtained from bagasse lignin’s hydrogenolysis.

On treatment of bagasse lignin with Pd-Fe_3_O_4_ and excess HCOONa in a mixture of ethanol and water at 150 °C for 6 h in an autoclave, a brown solution was obtained. After acidification, the products were extracted with ethyl acetate, a claybank soluble solution was obtained. The obtained monomers product’s solution was analyzed by GCMS. Figure 5 shows the TIC gas chromatogram of hydrogenolysis products from a bagasse lignin. As Figure 5 shows, a series of monomeric products can be observed. The individual peaks appeared in the GCMS profile were identified by comparing their retention times and mass spectra with those of the corresponding standards available commercially, or obtained by independent synthesis (for details, see the supplementary information). Eventually, 11 monomeric products were identified and the structure of these products are shown in Figure 6.

Small compounds obtained from bagasse lignin retain their aromatic character and aromatic hydroxyl groups, and these monomers fall into three catalogues according to their aromatic rings (p-hydroxyphenyl, guaiacyl, and syringyl): H1~H3, G1~G3, and S1~S5. Among these ten monomers, it is obvious that H3 monomer and G3 monomer were derived from p-coumaric and ferulic acids in the lignin. The yields of monomeric products were measured by GC using dodecane as the internal standard (IS) showed in Figure 5. In the current work, about 20.0 wt% monomer yield was achieved. These results indicate that the Pd-Fe_3_O_4_-catalyzed hydrogenolysis of bagasse lignin was promising and competitive.

### 3.3. Recyclability of Pd-Fe_3_O_4_

**Figure 7:**
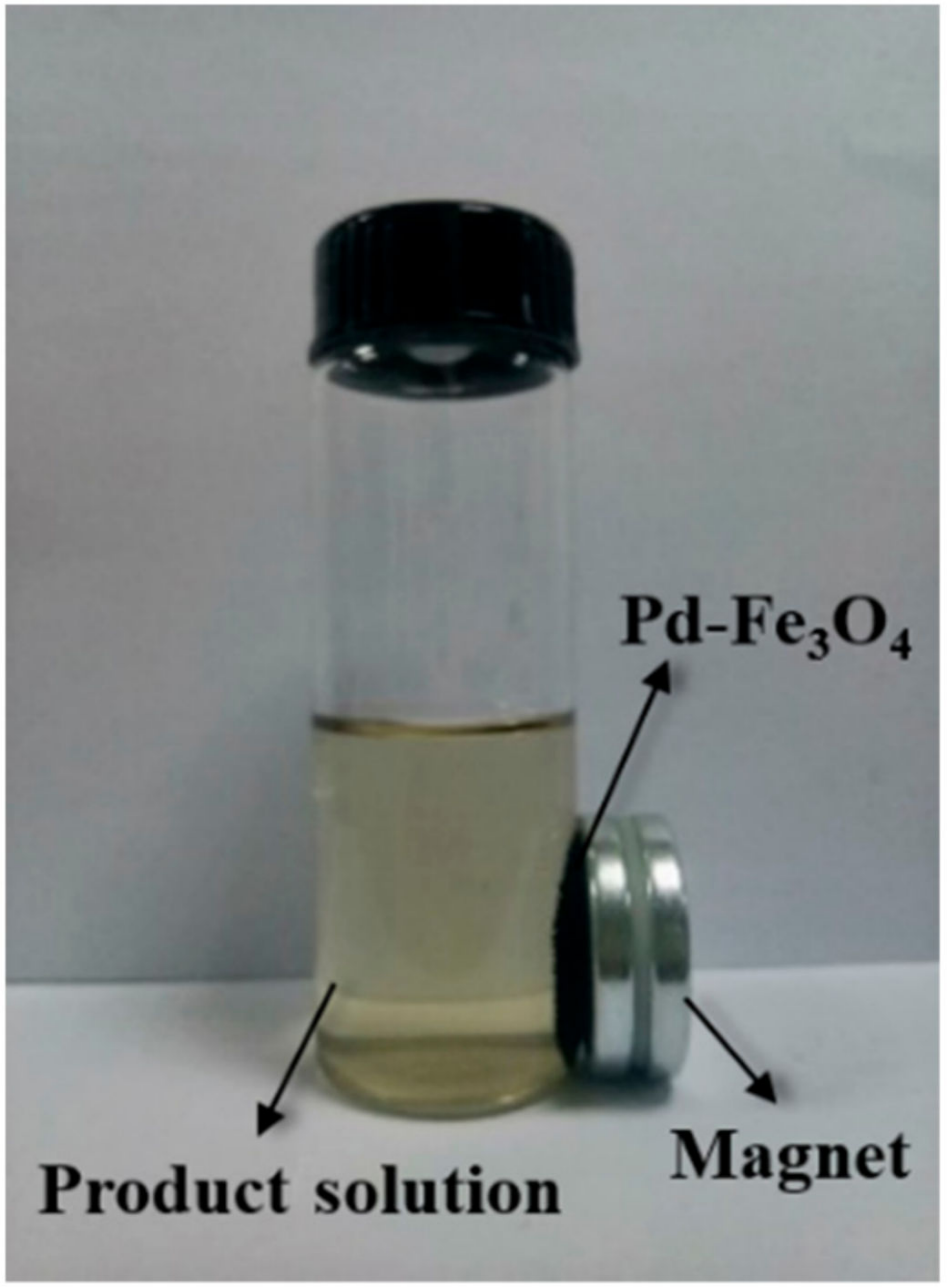
Separation of Pd-Fe_3_O_4_ catalyst from reaction mixture by an external magnet.

The convenience in separation and recyclability of metal catalysts are important for potential applications in industry. In the current study, the spent catalyst was separated by magnet conveniently. By using an external magnet to retain the Pd-Fe_3_O_4_ catalyst on the bottom, the reaction solution can be dumped conveniently as shown in Figure 7. After simple washing by water and ethanol, the collected catalyst was directly used in the following reaction.

**Figure 8:**
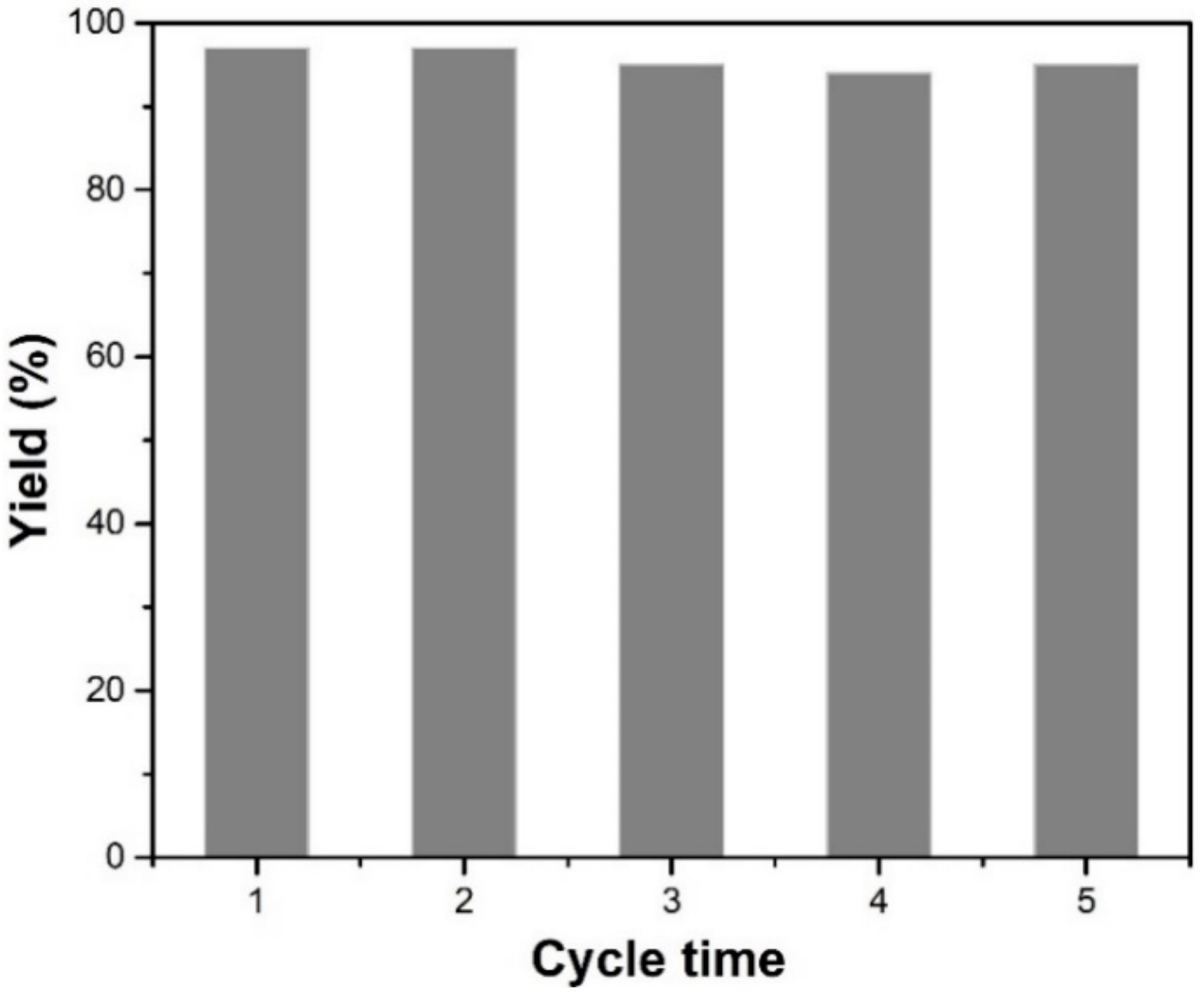
Yields of hydrogenolysis product from lignin model compound using recycled Pd-Fe_3_O_4_.

The collected and washed catalyst was directly used in the next reaction. As shown in Table 1 and Figure 8, the yield of monomeric product 2 from the dimeric model compound released by hydrogenolysis retained at 95% after five runs, demonstrating the high stability and efficiency of the Pd-Fe_3_O_4_ catalyst.

## 4. Conclusions

A magnetic catalyst (Pd-Fe_3_O_4_) was developed for the catalytic hydrogenolysis of β-O-4 bond in lignin. SEM, XRD, and XPS analyses indicated that Pd^0^ is uniformly deposited on the surface of the magnetic Fe_3_O_4_ nano-particles. The magnetic Pd-Fe_3_O_4_ catalyst demonstrated good performance in the hydrogenolysis of the model compound and bagasse lignin. More importantly, the Pd-Fe_3_O_4_ can be separated conveniently by a magnet and be reused at least five times retaining high product yields. Therefore, this magnetic Pd-Fe_3_O_4_ catalyst with excellent performances in lignin hydrogenolysis would be a good alternative to the traditional Pd/C used in lignin depolymerization and be widely applied for biomass utilizations.

## Figures and Tables

**Table 1:** Reusability of Pd-Fe_3_O_4_ for catalytic hydrogenolysis reaction in same react condition.

Cycle Times	Substrate	Catalyst	Solvent	Temperature	Time	Yield %
1	Dimer	Pd-Fe_3_O_4_	Ethanol	150 °C	6 h	97
2	Dimer	Pd-Fe_3_O_4_	Ethanol	150 °C	6 h	97
3	Dimer	Pd-Fe_3_O_4_	Ethanol	150 °C	6 h	95
4	Dimer	Pd-Fe_3_O_4_	Ethanol	150 °C	6 h	94
5	Dimer	Pd-Fe_3_O_4_	Ethanol	150 °C	6 h	95

## Supplementary Materials

The following are available online at http://www.mdpi.com/2073-4360/10/10/1077/s1, Figure S1: The NMR pictures of three identified monomers’ standard samples by independent synthesis: (a) G2, (b) monomer 1, (c) S5, Figure S2: The HSQC picture of bagasse lignin extracted, Figure S3: Examples of major linkages in bagasse lignin.
