# Supplementary material for: High-Efficient and Recyclable Magnetic Separable Catalyst for Catalytic Hydrogenolysis of β-O-4 Linkage in Lignin
Source: Polymers (Basel). 2018 Sep 28;10(10):1077. doi: 10.3390/polym10101077 (PMC6404071; doi:10.3390/polym10101077)
Supplement: Supplementary file 1 [file polymers-10-01077-s001.pdf]

Supplementary materials:

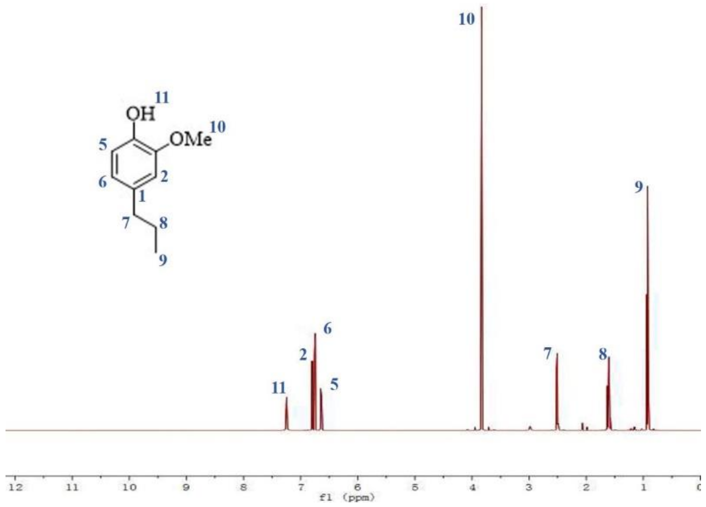

(**a**)

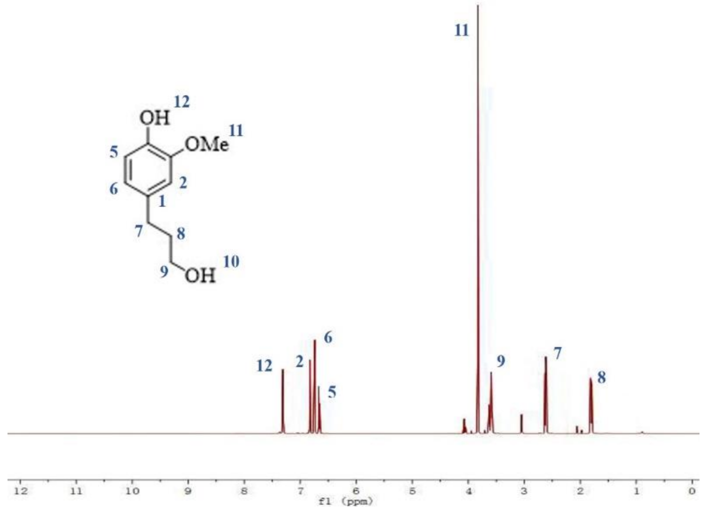

(**b**)

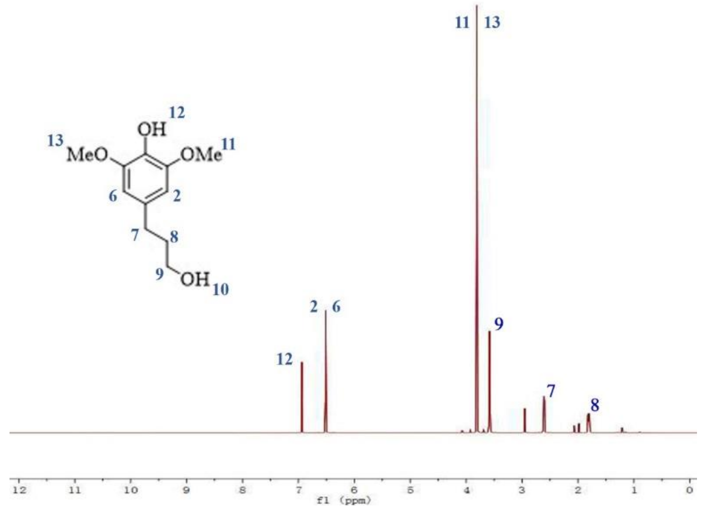

(**c**)

Figure S1. The NMR pictures of three identified monomers' standard samples by independent synthesis: (**a**) G2, (**b**) monomer 1, (**c**) S5.

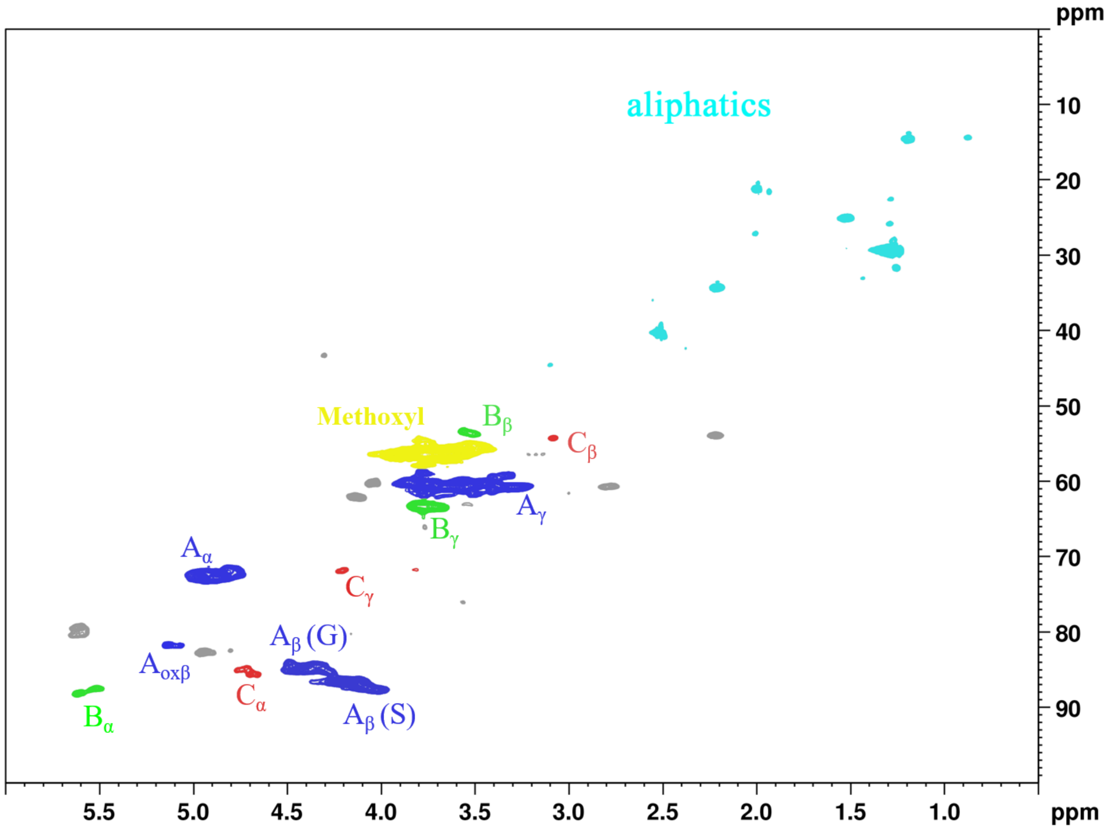

Figure S2. The HSQC picture of bagasse lignin extracted.

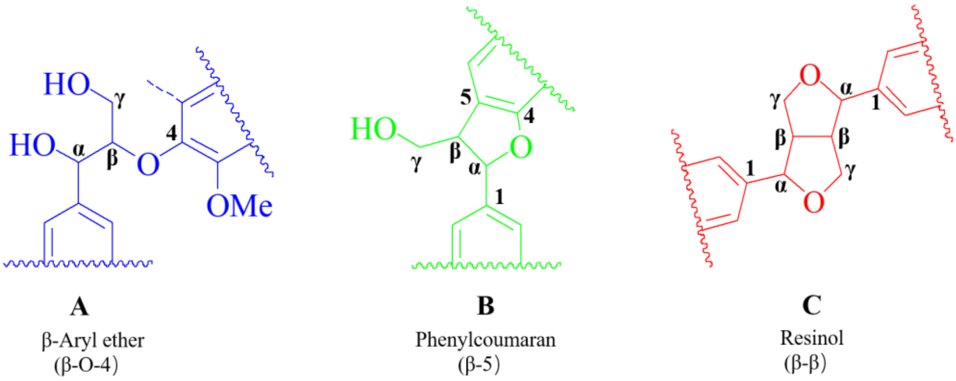

Figure S3. Examples of major linkages in bagasse lignin.
